# Supplementary material for: Wild gut microbiome suppresses the potentially opportunistic pathogen Aeromonas in medaka under domesticated rearing conditions
Source: Anim Microbiome. 2025 Oct 1;7:98. doi: 10.1186/s42523-025-00462-4 (PMC12486508; doi:10.1186/s42523-025-00462-4)
Supplement: Supplementary file 1 — Supplementary Material 1 [file 42523_2025_462_MOESM1_ESM.docx]

**Supplementary Information**

**Wild gut microbiome suppresses the potentially opportunistic pathogen *Aeromonas* in medaka under domesticated rearing conditions**

Keisuke Kawano^1,2†^, Kai Kawabe^1^, Yuki Sano^1††^, Tomoyuki Hori^3^, Minoru Kihara^2^, Yoshitomo Kikuchi^1,4^, Hideomi Itoh^4*^

^1^Graduate School of Agriculture, Hokkaido University, Sapporo, Hokkaido 060-8589, Japan

^2^School of Biological Sciences, Tokai University, Sapporo, Hokkaido 005-8601, Japan

^3^Environmental Management Research Institute, National Institute of Advanced Industrial Science and Technology (AIST) Tsukuba West, Tsukuba, Ibaraki 305-8569 Japan

^4^Bioproduction Research Institute, National Institute of Advance Industrial Science and Technology (AIST) Hokkaido, Sapporo, Hokkaido 062-8517, Japan

^†^Present address: Graduate School of Agriculture, Kyoto University, Sakyo-ku, Kyoto 606-8502, Japan

^††^Present address: Research Center, JAPAN NUTRITION Co., Ltd., Nasu-Shiobara, Tochigi 325-0103, Japan

*Correspondence:

Keisuke Kawano (kkawano.thku@gmail.com)

Hideomi Itoh (hideomi-itou@aist.go.jp)

**Supplementary Methods: Sampling and analysis of other fish species**

To evaluate whether the dominance of *Aeromonas* observed in domesticated medaka is a general phenomenon among ornamental fishes reared under similar indoor conditions, we included three other small fish species in our analysis—guppy (*Poecilia reticulata*), goldfish (*Carassius auratus*), and zebrafish (*Danio rerio*). These fish were housed in separate containers under the same indoor conditions as the D8 medaka. Dissection, DNA extraction, and gut microbiome analysis were conducted using the same procedures described for medaka in the main text.

**Supplementary Result: *Aeromonas* abundance in other domesticated fish species**

We additionally analyzed the gut microbiota of guppy, goldfish, and zebrafish from the same pet store as the D8 group. The relative abundance of *Aeromonas* in the gut microbiota was 5.0%–10.9% in guppy (mean ± SD = 7.5% ± 2.2%, n = 6), 10.4%–92.3% in goldfish (mean ± SD = 49.6% ± 20.6%, n = 6), and 15.0%–27.9% in zebrafish (mean ± SD = 17.0% ± 5.8%, n = 6) (Supplementary Fig. S4).**Supplementary figures:**

**Fig. S1** Specific bacterial groups in the gut microbiome of domesticated (D) or wild (W) groups. (A) Specific bacterial groups in D or W groups identified via LEfSe analysis. The top 10 genera with the highest LDA scores for D or W groups are shown. (B) Proportion of these specific bacterial groups in the gut microbiomes of each medaka.

**Fig. S2** Effect of antibiotics treatment on microbial abundance in the medaka gut, measured by colony-forming units (CFUs) on the 1.5% agar plates with three different media: Reasoner's 2A broth (R2A), Nutrient Broth (NB), and Luria-Bertani (LB) broth. Asterisks indicate significant differences (*p* < 0.05, Mann–Whitney U-test).


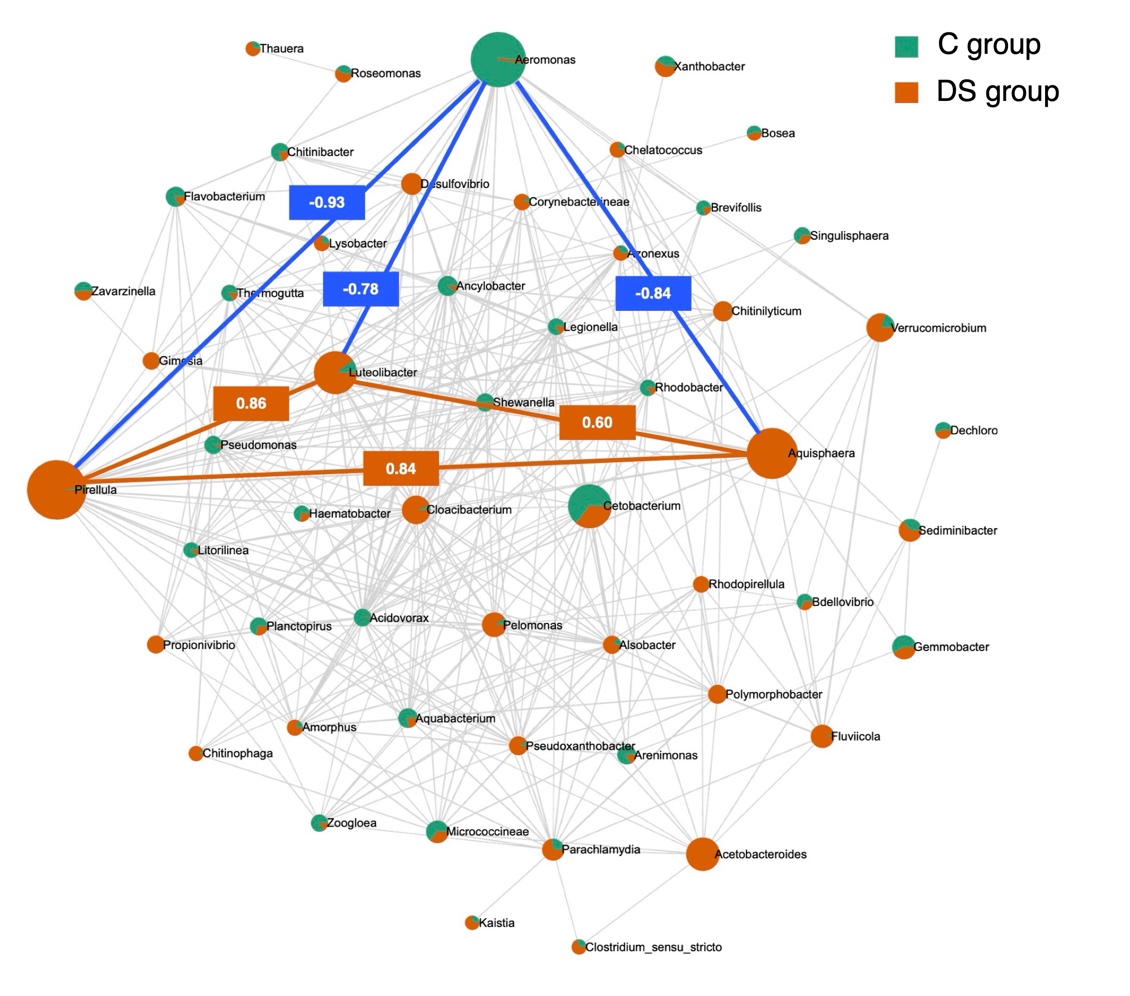


**Fig. S3** Correlation network of gut microbiome genera in Rearing Experiment 2. Each pie chart node represents a bacterial genus, with colors indicating the relative abundance in different groups: green for the C group and red for the DS group. Edges represent correlations between genera, with correlations involving *Aeromonas*, *Pirellula*, *Aquisphaera*, and *Luteolibacter* highlighted in bold. Edge colors indicate correlation direction: red for positive correlations and blue for negative correlations. Numbers above the edges show the correlation coefficients.

**Fig. S4** Proportion of *Aeromonas* in the gut microbiomes of domesticated fish species. Guppy, goldfish, and zebrafish examined in this experiment were maintained in the same aquarium shop where D8 medaka were purchased.

**Supplementary Tables:**

**Supplementary Table S1 | Potential confounding factors considered in GLMMs.**

Provided as a separate Excel sheet.

**Supplementary Table S2 | Identification and haemolytic activity of bacterial strains isolated from medaka gut in the D8 group.**

**Supplementary Table S3 | Dominant members of the gut microbiome in medaka reared for one week with sediment from the site where W6 group were collected.**

**Supplementary Table S4 | Dominant members of the gut microbiome in medaka reared for one week with sediment from the site where W12 group were collected.**

**Supplementary Table S5 | Dominant members of the gut microbiome in medaka reared for one week with sediment from the site where W14 group were collected.**

**Supplementary Table S6 | Dominant members of the gut microbiome in medaka reared for one week with sediment from the site where W15 group were collected.**

**Supplementary Table S7 | Dominant bacterial members of the rearing water used for Step 3 in Rearing Experiment 2.**
